# Association Between Cannabis Use and Brain Structures: A Mendelian Randomization Study

**DOI:** 10.7759/cureus.65922

**Published:** 2024-08-01

**Authors:** Juan Li, Zhao Yin, Zeming Yu, Jiannan Li, Lu Yang

**Affiliations:** 1 Department of Anesthesiology, The Sixth Medical Center of People's Liberation Army (PLA) General Hospital, Beijing, CHN; 2 Department of Cardiology, People's Liberation Army (PLA) Strategic Support Force Characteristic Medical Center, Beijing, CHN

**Keywords:** mendelian randomization, subcortical structure, cortical structure, cannabis use disorder, lifetime cannabis use

## Abstract

Background

Observational studies suggested that cannabis use was associated with alternation of brain structures; however, as subjected to confounding factors, they were difficult to make causal inferences and direction determinations. In this study, a two-sample Mendelian randomization (MR) analysis was employed to examine the potential causal association between cannabis use and brain structures.

Methods

The genome-wide association studies (GWAS) data for lifetime cannabis use (LCU), cannabis use disorder (CUD), and brain cortical and subcortical structures were utilized in this study. Cortical structures were divided into 34 distinct gyral-defined regions with surface area (SA) and thickness (TH) measured. Subcortical structures encompassed volumes from seven specified regions. The primary estimator used in our analysis was inverse-variance weighted (IVW), complemented by MR-Egger and weighted median methods to enhance the robustness of the results. The Cochran’s Q test, funnel plots, and MR-Egger intercept tests were used to detect heterogeneity and pleiotropy.

Results* *

No causal relationship was detected between LCU and global cortical SA or TH. However, at the regional cortex level, LCU was associated with decreased TH in the fusiform (β = -0.0168 mm, SE = 0.00581, P = 0.0039) and lateral occipital (β = -0.0141 mm, SE = 0.00531, P = 0.0079) regions, while increasing TH in the postcentral region (β = 0.0093 mm, SE = 0.00445, P = 0.0374). At the subcortical level, LCU was found to increase the brainstem volume (β = 0.224 mm^3^, SE = 0.09, P = 0.0128). CUD did not show any causal association with brain structure at either cortical or subcortical levels. Nonetheless, after applying multiple comparison corrections, the P values for the MR analysis of causal relationships between cannabis use and these brain structures did not meet the significance threshold.

Conclusion* *

The evidence for cannabis use causally influencing brain structures is insufficient.

## Introduction

Cannabis is a widely produced and consumed illicit substance in the world [1]. As more legal markets emerged, its prevalence gradually increased. Occasional cannabis use can progress to frequent use, abuse, and dependence [2]. In addition, there may also be a proportion of the population who are administered cannabis as a daily medicine [3]. It has been reported that cannabis use could lead to cognitive deficits and its misuse has been associated with a range of neuropsychiatric disorders [4-6]. Brain structures, particularly the cerebral cortex, are considered valuable neuroimaging indicators for predicting future cognitive declines [7]. Altered macroscale brain structure is reported to be associated with psychopathology and could represent a mechanistic link between cannabis-associated neurotoxicity and health outcomes [8]. Comprehension of the relationship between cannabis use and the alternations of brain structure would be important for effective prevention and intervention and, therefore, of paramount relevance to public health. 

A growing body of literature suggests that cannabis intake can induce brain structural alternation. Previous magnetic resonance imaging studies in regular cannabis users have reported altered grey matter volume in brain regions, including the prefrontal cortex, putamen, and hippocampus [9]. In another study, Knodt et al. also observed that long-term cannabis users had a thinner cortex, smaller subcortical gray matter volumes, and higher machine learning-predicted brain age than non-users [10]. However, the causality of these relationships remains unclear, as previous studies have inevitably faced challenges, including insufficient sample sizes, difficulties in controlling confounding factors, and establishing clear temporal sequencing of events [11]. Although randomized controlled trials (RCTs) could avoid these biases, performing them is unfeasible and unethical.

Faced with this challenge, mendelian randomization (MR) analysis, a method from genetic epidemiology, was used in this study. MR is an approach that utilizes genetic variants, typically single nucleotide polymorphisms (SNPs), as instrumental variables (IVs) to infer causality between exposures and outcomes. These SNPs are selected based on their association with the exposure of interest and are assumed to be randomly assigned according to the Mendelian inheritance principles [12]. Thus,* *in contrast to traditional observational studies, MR analysis has the potential to reduce confounding biases and offer evidence of a quality comparable to that of randomized controlled trial (RCT) studies [12]. In this study, we used the two-sample MR method to increase the understanding of the causal relationship between cannabis use and brain structures. The summary of genome-wide association studies (GWAS) data on cannabis was utilized to predict genetic alteration in the cortical surface area, thickness, and subcortical volumes. Following this, certain sensitivity analyses were employed to address the heterogeneity and pleiotropy effects, thereby validating the robustness of the causal relationships.

## Materials and methods

Exposure and outcome data source

The GWAS data on lifetime cannabis use (LCU), defined as any cannabis consumption across an individual's lifespan, were obtained from a study by Pasman et al., which included an investigation of 162,082 individuals of European ancestry [13]. The meta-analysis of GWAS studies consisted of data from the International Cannabis Consortium (N=35,297, 42.8% cases, and 55.5% females) and UK Biobank (N=126,785, 22.3% cases and 56.3% females). Genotyping was performed on various genotyping platforms, and standard quality control checks were performed before imputation. Details regarding ethical approval and informed consent can be found in the original paper [13]. The summary-level GWAS data for cannabis use disorder (CUD) were obtained from a meta-analysis of GWAS studies of 357,806 individuals of European ancestry (14,080 cases and 343,726 controls) [14]. The data consisted of three sources, including the Psychiatric Genomics Consortium (N= 15,293, 34.6% cases), Lundbeck Foundation Initiative for Integrative Psychiatric Research (N= 56,084, 4.9% cases), and deCODE (N = 286,429, 2.1% cases) [14].In these research consortia, CUD was diagnosed based on the criteria outlined in the International Classification of Diseases-10 (ICD-10) or the Diagnostic and Statistical Manual of Mental Disorders-IV (DSM-IV), characterized by an individual's persistent use of cannabis despite significant social or health-related adverse consequences [14].

The summary-level GWAS data for the cerebral cortical structure were obtained from the Enhancing Neuro Imaging Genetics through Meta-Analysis (ENIGMA) Consortium [7]. The analyses included GWAS of brain MRI data of 51,665 individuals (predominantly of European ancestry) from 60 cohorts worldwide. Measurements of the cortical surface area (SA) and mean thickness (TH) were conducted globally for the entire brain and 34 specific brain regions based on the Desikan-Killiany atlas. SA was quantified at the boundary between grey and white matter, while the TH was determined as the average distance between the white matter and pial surfaces. To account for the distinct genetic influences of each brain region, the GWAS dataset was corrected using globally measured cortical SA and mean TH as covariates. We utilized the global-weighted GWAS data of cerebral cortex structures for the subsequent MR analysis [7]. The spatial localization of these structures is detailed in Table 1.

**Table 1:** Anatomical information of different cortical structures

Lobe	Area
Frontal	Frontal Pole, Medial Orbitofrontal, Lateral Orbitofrontal, Rostral Anterior Cingulate, Caudal Anterior Cingulate, Superior Frontal, Rostral Middle Frontal, Pars Orbitalis, Pars Triangularis, Pars Opercularis, Caudal Middle Frontal, Paracentral, Precentral
Parietal	Postcentral, Precuneus, Superior Parietal, Supramarginal, Inferior ParietalInferior Parietal, Posterior Cingulate, Isthmus Cingulate
Temporal	Insula, Entorhinal, Parahippocampal, Fusiform, Temporal Pole, Inferior Temporal, Middle Temporal, Superior Temporal, Banks of the Superior Temporal Sulcus, Transverse Temporal
Occipital	Lingual, Pericalcarine, Cuneus, Lateral Occipital

The summary-level GWAS data for subcortical brain structure were obtained from a meta-analysis of MRI studies involving 38,851 individuals from 53 cohorts with participants of primarily European ancestry. The data sources included the Cohorts of Heart and Aging Research in Genomic Epidemiology (CHARGE), ENIGMA, and UK Biobank [15]. The seven subcortical brain structures, including accumbens, amygdala, caudate, pallidum, putamen, thalamus, and brainstem, were characterized by the mean volume of bilateral hemispheres, excluding the brainstem where total volume was utilized. An unrestricted summary data with slightly smaller sample sizes (excluding the AGES, ARIC, CHS, and FHS cohorts) were used in this study [15]. 

Instrumental variables (IVs)

To guarantee the validity of MR analysis, IVs were selected based on the following criteria: 1) they must be closely associated with the exposure; 2) they should be independent of confounders that might bias the relationship between the exposure and the outcome; 3) they must influence the outcome exclusively through its effect on the exposure, thus ensuring a direct causal pathway. In this study, genetic instruments associated with the phenotype LCU and CUD were selected at a GWAS-correlated P value < 5x10^-7^ and linkage disequilibrium clumping at r^2^ < 0.001, clumping distance = 10000 kb. To prevent weak-tool bias in MR analysis, the F statistic of instruments was used to evaluate the strength of associations between SNPs and exposure as previously described [11]. When the F-values were above ten, the SNPs were considered strong instruments and used in the following MR analysis [16]. To prevent the effects of confounders on the following MR analysis, we checked each candidate SNP in PhenoScanner V2 (http://www.phenoscanner.medschl.cam.ac.uk/) [17]. SNPs associated with potential outcome risk factors, including BMI, mental disorders, tobacco smoking, insomnia, alcohol intake, and education attainment, were removed for the following MR analysis. Then, SNPs with a palindromic strand (A/T, C/G alleles) and the underlying outliers identified by the MR pleiotropy residual sum and outlier (MRPRESSO) test were removed before MR analysis. 

TwoSampleMR analysis

After removing the cortical and subcortical structure-related SNPs with a threshold of 5x10^-8^, harmonization was performed to rule out strand mismatches and to ensure alignment of effect sizes. Then, the effects of LCU or CUD on brain structure-related traits were estimated by performing multiplicative random effects inverse-variance weighted (IVW), MR-Egger, and weighted median methods. Although MR-Egger and weighted median methods are less efficient, they could offer more robust estimates across a wider range of scenarios. To enhance the reliability of our conclusions, we primarily relied on the IVW estimates, whereas additionally utilized the MR-Egger and weighted median results to strengthen the IVW outcomes. We considered the results as significant when the IVW results were significant, and both MR-Egger and weighted median results aligned directionally with IVW.

Sensitivity analysis

**Figure 1:**
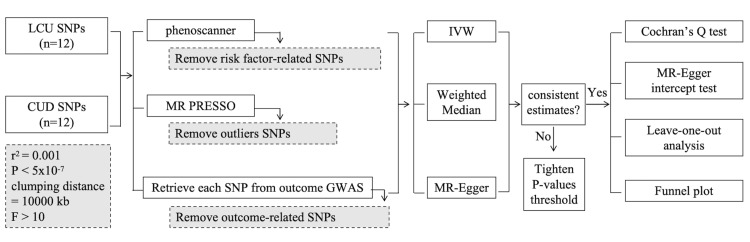
The flowchart of TwoSampleMR analysis revealing the causality between cannabis use and brain structures LCU: lifetime cannabis use; CUD: cannabis use disorder; SNPs: single nucleotide polymorphisms; MR PRESSO: MR pleiotropy residual sum and outlier; GWAS: genome-wide association study; IVW: inverse-variance weighted.

To prevent the influence of heterogeneity and pleiotropy on MR analysis, we applied several sensitivity analysis tests. The Cochran’s Q test was employed to identify heterogeneity, and if heterogeneity was detected as less than 0.05, only multiplicative random-effects IVW was used in this MR analysis. We utilized funnel plots to assess the probable directional pleiotropy and MR-Egger intercept tests to assess the horizontal pleiotropy. Then the leave-one-out analyses were conducted to determine if the IVW estimate was influenced by any single SNP [18]. The study flowchart is depicted in Figure 1.

Statistical analysis

All analyses were conducted using R (version 4.3.1, https://www.r-project.org/) with the TwoSampleMR (version 0.5.7, https://github.com/MRCIEU/TwoSampleMR) and MR PRESSO (version 1.0, https://github.com/rondolab/MR-PRESSO) packages. For the cortical regional-level tests, we conducted the two-sample MR analyses 68 times, with the significance threshold adjusted to 0.05/68 (7.35×10^-4^) using the Bonferroni method. The global-level tests, which involved both SA and TH directions, considered the corrected significant P-value as 0.05/2 (0.025). For the subcortical-level test, considering the seven MR estimates, a significant P-value was defined as 0.05/7 (7.14x10^-3^). A P-value less than 0.05 indicated nominal significance.

Data availability

GWAS data utilized in this study are publicly available and can be found in online repositories. The summary statistics data for the lifetime cannabis use are available at https://www.ru.nl/bsi/research/group-pages/substance-use-addiction-food-saf/vm-saf/genetics/international-cannabis-consortium-icc/ [13]. The cannabis use disorder GWAS data by Johnson et al. are available at https://pgc.unc.edu/for-researchers/download-results/ [14]. The cortical and subcortical GWAS data by Grasby et al. [7] and Satizabal et al. [15] could be accessed via https://enigma.ini.usc.edu/research/download-enigma-gwas-results/.

## Results

Selection of instrumental variables

In total, 12 SNPs were selected for LCU and 12 SNPs were selected for CUD. Then, five SNPs (rs1154693; rs1368740; rs9919557; rs10883796; rs17761723) were removed for LCU and five SNPs (rs719504; rs1392816; rs7783012; rs11783093; rs719012) were removed for CUD as they were associated with potential outcome risk factors. One SNP (rs17514242) was removed for CUD and one SNP (rs9578502) was removed for LCU with a palindromic strand (A/T, C/G alleles). One SNP (rs75448266) was removed for LCU as related to cortical and subcortical structure GWAS data. Finally, five SNPs for LCU and six SNPs for CUD were used in this MR analysis. Details on instrumental variables and F-statistic values are presented in Tables 2, 3.

**Table 2:** Details of five genome-wide significant SNPs for LCU used for MR analysis SNP: single nucleotide polymorphism; LCU: lifetime cannabis use; EA: effect allele; OA: other allele; EAF: effect allele frequency; BETA: regression coefficient; SE: standard error; F: F-statistic values

SNP	EA	OA	EAF	BETA	SE	P	N	F	R2
rs1816793	T	C	0.3694	0.0492	0.0093	1.22x10^-7^	162082	182.9913397	0.001127745
rs353253	A	G	0.3319	-0.1357	0.0267	3.73x10^-7^	35297	290.6115352	0.008166546
rs4099556	A	G	0.8242	0.0699	0.012	5.71x10^-9^	162082	229.8161866	0.001415911
rs9435794	T	C	0.7091	-0.0554	0.0103	7.51x10^-8^	162082	205.4851349	0.001266195
rs9972414	A	G	0.2899	0.0537	0.0099	5.82x10^-8^	162082	192.6600419	0.001187261

**Table 3:** Details of six genome-wide significant SNPs for CUD used for MR analysis SNP: single nucleotide polymorphism; CUD: cannabis use disorder; EA: effect allele; OA: other allele; EAF: effect allele frequency; BETA: regression coefficient; SE: standard error; F: F-statistic values

SNP	EA	OA	EAF	BETA	SE	P	N	F	R2
rs11715758	A	G	NA	-0.0935	0.0175	9.15x10^-8^	342452	28.54595573	8.33511x10^-5^
rs72818514	T	C	NA	-0.1828	0.0342	9.04X10^-8^	355548	28.56917687	8.03465x10^-5^
rs553920	T	C	NA	0.104	0.0198	1.50x10^-7^	353969	27.58886564	7.79358x10^-5^
rs9787909	A	C	NA	0.1137	0.0225	4.34x10^-7^	354449	25.53603369	7.20395x10^-5^
rs1509514	A	G	NA	-0.0853	0.0167	3.26x10^-7^	356895	26.08931559	7.30959x10^-5^
rs17271123	T	G	NA	0.1284	0.0252	3.48x10^-7^	291017	25.96127283	8.92014x10^-5^

TwoSampleMR analysis results

**Figure 2:**
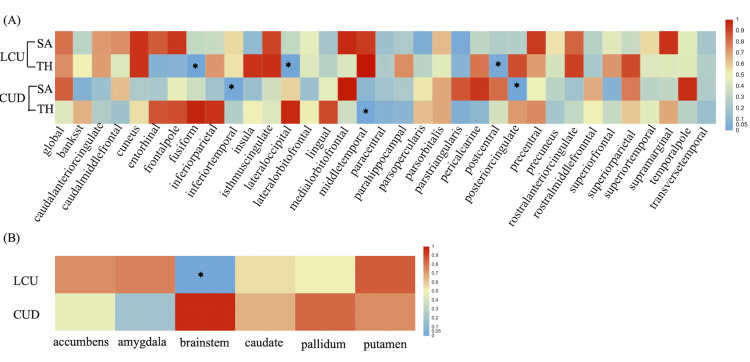
IVW results from MR analysis of LCU and CUD on brain cortical and subcortical structures IVW: inverse-variance weighted; LCU: lifetime cannabis use; CUD: cannabis use disorder; SA: surface area; TH: thickness; The asterisk indicates a nominal significant result with a p < 0.05 (*: P<0.05).

For global cortex structure, LCU did not exhibit a causal association with the global cortex SA and TH (β_SA_ = 190.914 mm^2^, SE_SA_ = 710.453, P_SA_ = 0.788; β_TH_ = 0.0027 mm, SE_TH_ = 0.008, P_TH_ = 0.7363). Similarly, CUD did not show a causal association with the global cortex SA and TH either (β_SA_ = -109.293 mm^2^, SE_SA_ = 509.512, P_SA_ = 0.83; β_TH_ = 0.0026 mm, SE_TH_ = 0.0032, P_TH_ = 0.425). No pleiotropy or heterogeneity was detected. For regional cortex structure, there were several suggestive gyri, including fusiform, inferior temporal, lateral occipital, middle temporal, postcentral, and posterior cingulate, potentially influenced by cannabis use (P_ivw_ < 0.05). Details are presented in Figure 2.

LCU was found to potentially decrease the TH of the fusiform (β = -0.0168 mm, SE = 0.00581, P = 0.0039) and lateral occipital (β = -0.0141 mm, SE = 0.00531, P = 0.0079) but increase the TH of the postcentral (β = 0.0093 mm, SE = 0.00445, P = 0.0374). Consistency analyses with weighted median and MR Egger methods further confirmed the aforementioned results, with sensitivity analysis excluding heterogeneity and pleiotropy issues as well. Details are presented in Table 4.

**Table 4:** Nominal significant results from MR analysis of LCU on brain cortical and subcortical structures LCU: lifetime cannabis use; IVW: inverse-variance weighted; MR-PRESSO: MR pleiotropy residual sum and outlier; BETA: regression coefficient; SE: standard error; TH: thickness; WM: weighted median.

Exposure	Outcomes	Methods	P	BETA	SE	Cochran’s Q	MR-Egger intercept	MR-PRESSOR
LCU	TH of fusiform	IVW	0.0039	-0.0168	0.0058	0.2648	0.5574	0.4378
		MR-Egger	0.1873	-0.0259	0.0152			
		WM	0.0175	-0.0177	0.0075			
	TH of lateral occipital	IVW	0.0079	-0.0141	0.0053	0.2395	0.3236	0.352
		MR-Egger	0.1129	-0.0272	0.0122			
		WM	0.00196	-0.0184	0.0059			
	TH of postcentral	IVW	0.0374	0.0093	0.0045	0.4512	0.497	0.5037
		MR-Egger	0.8962	0.00156	0.011			
		WM	0.3289	0.00568	0.00581			
	Brainstem volume	IVW	0.0128	0.2240	0.0900	0.5258	0.6208	0.6515
		MR-Egger	0.3532	0.2518	0.2552			
		WM	0.0307	0.2556	0.1183			

However, the current level of evidence was insufficient, as after applying the Bonferroni correction, neither genetically predicted LCU nor CUD was causally associated with the alterations of brain structures. There was a nominally significant association between CUD and the SA of inferior temporal and posterior cingulate, as well as the TH of middle temporal (P_ivw_ < 0.05), but the consistency analysis was inadequate to support the inference, with the MR-Egger method showing an opposite direction. Details are presented in Table 5.

**Table 5:** Nominal significant results from MR analysis of CUD on brain cortical structures CUD: cannabis use disorder; IVW: inverse-variance weighted; MR-PRESSO: MR pleiotropy residual sum and outlier; BETA: regression coefficient; SE: standard error; SA: surface area; TH: thickness; WM: weighted median.

Exposure	Outcomes	Methods	P	BETA	SE	Cochran’s Q	MR-Egger intercept	MR-PRESSOR
CUD	SA of inferior temporal	IVW	0.0302	-21.636	9.984	0.669	0.319	0.698
		MR-Egger	0.5377	33.159	49.257			
		WM	0.0251	-26.998	12.057			
	SA of posterior cingulate	IVW	0.00271	-12.1071	4.0374	0.8116	0.5615	0.8416
		MR-Egger	0.9909	0.2427	19.9438			
		WM	0.0218	-11.9003	5.1878			
	TH of middle temporal	IVW	0.0277	-0.0083	0.0038	0.5272	0.2036	0.5692
		MR-Egger	0.3567	0.0193	0.0186			
		WM	0.2431	-0.0057	0.0049			

After tightening the P values threshold for genetic instruments for CUD to 5x10^-8^, there were only two SNPs (rs7783012, rs11783093) left, and these two SNPs are associated with insomnia, alcohol intake or tobacco smoking, which are the risk factors for the alternations of brain structures. Thus, the following analysis could not be conducted. For subcortex structure, LCU potentially increased the volume of the brainstem (β = 0.224 mm^3^, SE = 0.09, P = 0.0128), and the result was supported by consistency analysis of weighted median and MR Egger (Table 4). CUD had no causal relationship with the volume of seven subcortex structures.

**Figure 3:**
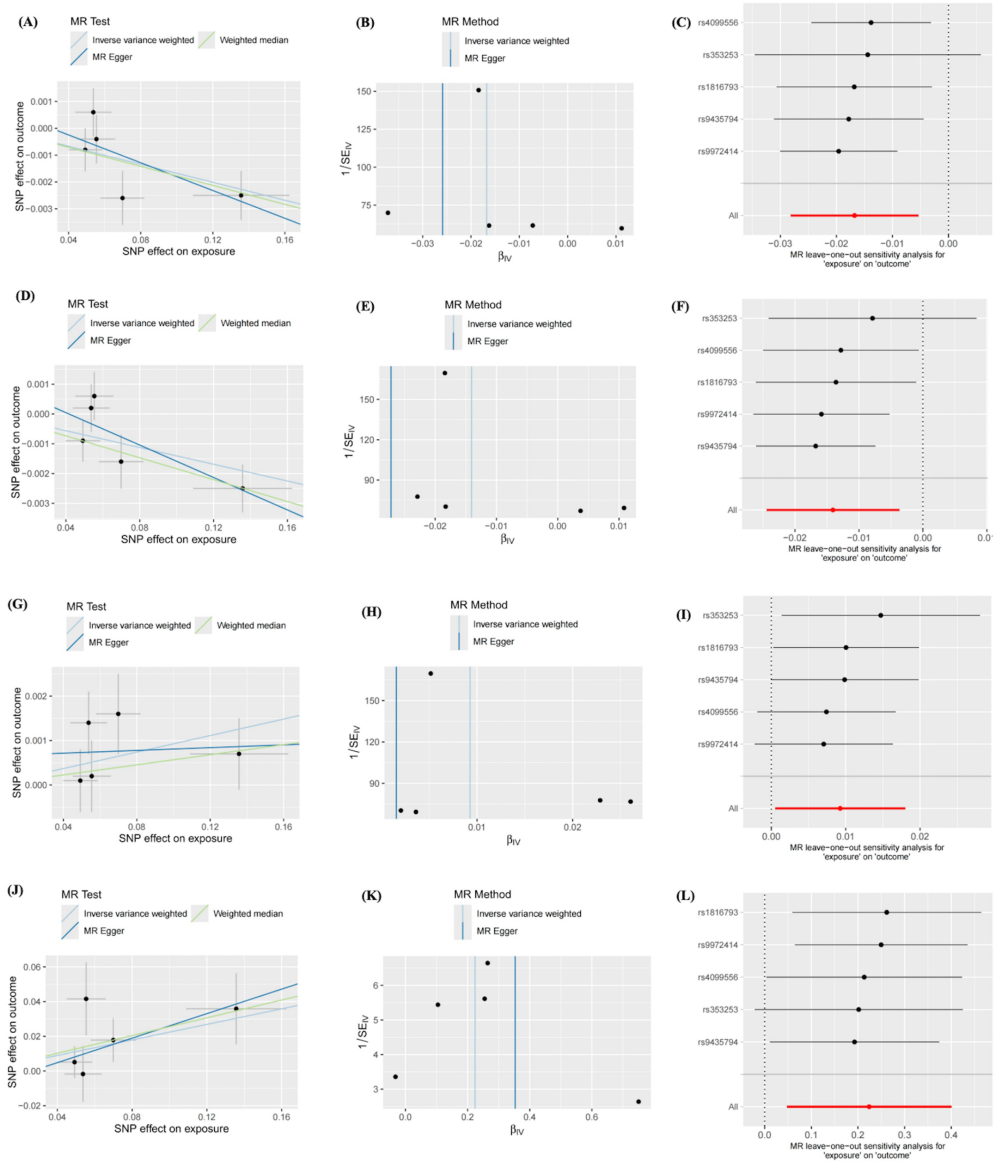
Sensitivity analyses of nominal significant estimates from LCU on brain structures A. Scatter plot of LCU effects on the TH of fusiform B. Funnel plot of LCU effects on the TH of fusiform C. Leave-one-out plot of LCU effects on the TH of fusiform D. Scatter plot of LCU effects on the TH of lateral occipital E. Funnel plot of LCU effects on the TH of lateral occipital F. Leave-one-out plot of LCU effects on the TH of lateral occipital G. Scatter plot of LCU effects on the TH of postcentral H. Funnel plot of LCU effects on the TH of postcentral I. Leave-one-out plot of LCU effects on the TH of postcentral J. Scatter plot of LCU effects on the volume of brainstem K. Funnel plot of LCU effects on the volume of brainstem L. Leave-one-out plot of LCU effects on the volume of brainstem LCU: lifetime cannabis use; TH: thickness

**Figure 4:**
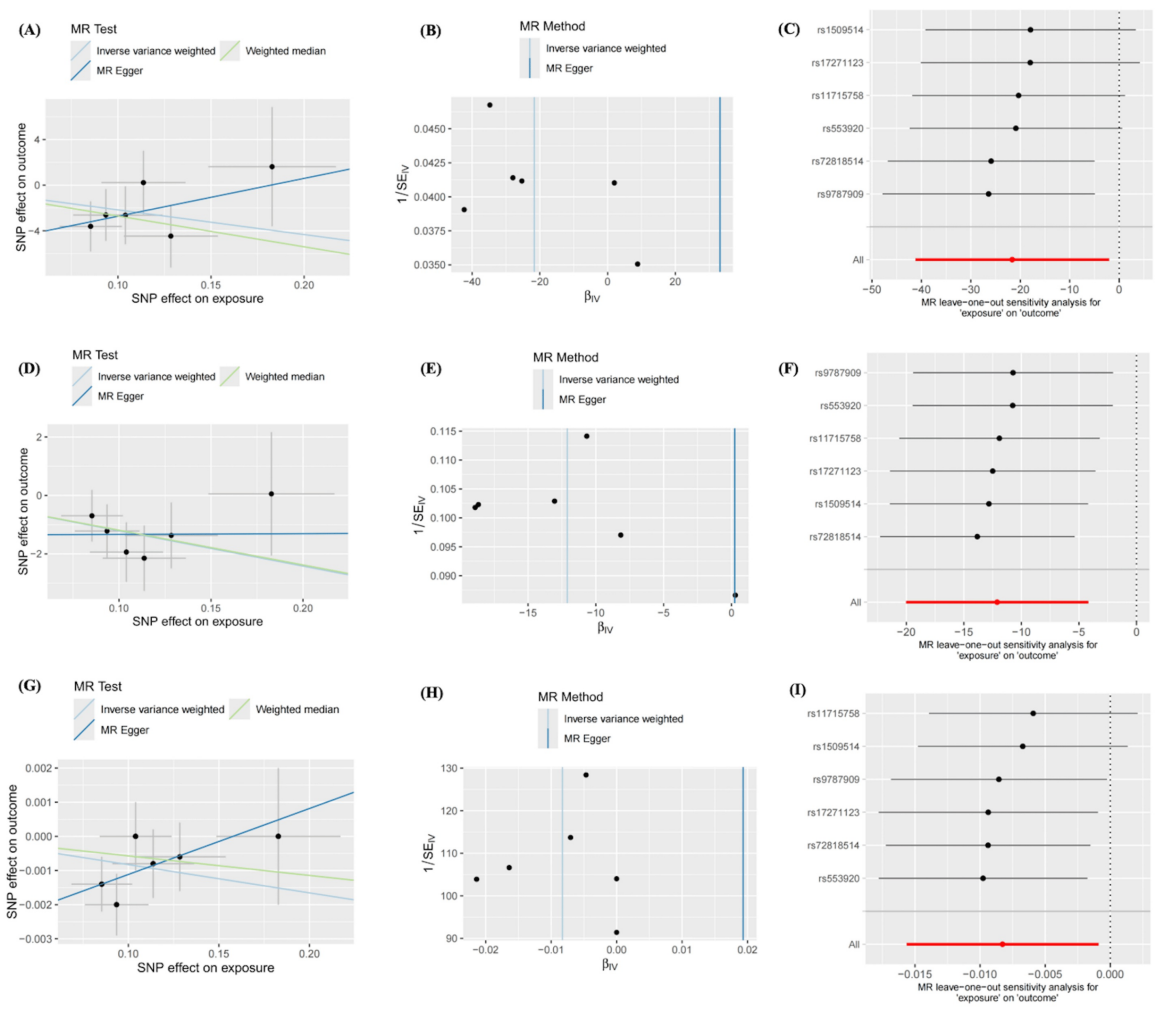
Sensitivity analyses of nominal significant estimates from CUD on brain structures A. Scatter plot of CUD effects on the SA of inferior temporal B. Funnel plot of CUD effects on the SA of inferior temporal C. Leave-one-out plot of CUD effects on the SA of inferior temporal D. Scatter plot of CUD effects on the SA of posterior cingulate E. Funnel plot of CUD effects on the SA of posterior cingulate F. Leave-one-out plot of CUD effects on the SA of posterior cingulate G. Scatter plot of CUD effects on the TH of middle temporal H. Funnel plot of CUD effects on the TH of middle temporal I. Leave-one-out plot of CUD effects on the TH of middle temporal CUD: cannabis use disorder; SA: surface area; TH: thickness

For all the nominal significant estimates, no heterogeneity or pleiotropy was detected. All Cochran’s Q test-derived P values and the P values for the MR-Egger intercept were greater than 0.05. Details are presented in Tables 4, 5. No outliers were identified from the MR-PRESSSO test, the leave-one-out sensitivity test, or the funnel plots. Detailed information on the scatter plots, leave-one-out analyses, and funnel plots is presented in Figures 3, 4.

## Discussion

In this Mendelian randomization (MR) study, we comprehensively assessed the causal relationship between cannabis use and brain structures, as predicted by genetic variants. While our findings did not provide significant evidence supporting a causal relationship between cannabis use and alterations in brain structures, we suggested careful consideration for cannabis users regarding the four brain regions including fusiform, lateral occipital, postcentral, and brainstem.

In previous studies, the effects of cannabis on brain structure were controversial. It has been reported that cannabis use could be associated with significant structural changes in the functional regions of the brain [19]. Subramaniam et al. have reviewed current findings on neuroimaging studies of adolescent cannabis users and indicated that cannabis use is associated with alterations in brain structure and function, especially in the regions that express high levels of the cannabinoid 1 receptors such as the prefrontal cortex, amygdala, hippocampus, cerebellum and limbic system [20]. However, it has also been reported that cannabis use did not affect cortical or subcortical morphologies [21]. These inconsistent findings could be explained by the presence of confounding factors included in these studies. Persistent cannabis use may lead to engagement in other mental disorders, like bipolar and schizophrenia, which in turn increase the alternations of brain structure [5,6]. Also, cannabis use is most commonly mixed with tobacco smoking and alcohol consumption, which are substances closely related to brain structures [22]. Thus, distinguishing the impact of cannabis from that of tobacco and alcohol on brain structures is challenging. As mental disorders, tobacco, and alcohol are related to both cannabis use and alterations in brain structures, it is essential to adjust for their effects when assessing the association between cannabis use and brain structures. Gillespie et al. tried to disentangle the putative impacts of cannabis on brain morphology from other comorbid substance use through exploratory analyses using mixed linear models, and they found that cannabis use was unrelated to any subcortical grey matter volumes [23]. Similarly, after critically controlling for alcohol use, gender, age, and other relevant confounders, a cross-sectional study indicated that there is no association between marijuana use and standard volumetric or shape measurements of subcortical structures [24].

In our study, we did not find a significant causal association between cannabis use and brain structure. However, it does provide evidence of nominally significant changes in the brain regions such as fusiform, lateral occipital, postcentral, and brainstem. We found that the thickness of the fusiform and the lateral occipital cortex was decreased after cannabis use. The fusiform and the lateral occipital gyri are high-level visual cortexes essential for visual recognition, performing complex functions such as recognizing objects, facial features, and motion [25]. They could integrate and analyze visual information along with auditory and other sensory information. These regions are linked to brain areas that support speech, executive functions, as well as visual memories [26]. It has been reported that higher levels of cannabis use were associated with smaller volumes in the fusiform gyrus [27]. Compared to the total volume change, cannabis was more prone to reduce the thickness of the fusiform gyrus [28]. Therefore, we venture to infer that cannabis use decreases the thickness of the fusiform and lateral occipital cortex to impair visual memory-related function processing.

The thickness of the postcentral gyrus and the volume of the brainstem were found to increase after cannabis use in our study. Postcentral is confirmed as a sensory cortical center, which participates in the function of somatosensory processing, particularly position sense [29]. The brainstem is the structure that connects the cerebrum of the brain with the spinal cord and cerebellum. It is responsible for many vital functions of life, such as breathing, consciousness, blood pressure, heart rate, and sleep [30]. It also provides the main motor and sensory nerve supply to the face and neck via the cranial nerves [30]. In our study, LCU increased the TH of the postcentral cortex and the volume of the brainstem, which were inconsistent with the results of a previous study. An observational study by James et al. reported that early cannabis use was associated with a greater reduction of white matter integrity in the brainstem and loss of grey matter density in postcentral gyrus in adolescent-onset schizophrenia patients [31]. Imaging studies have reported that individuals with an early age of cannabis use onset were more likely to demonstrate abnormalities in brain functions and structures [32]. Compared to later use onset, early cannabis use was associated with different brain morphology [33]. Besides, both schizophrenia and cannabis use have been associated with brain structural abnormalities. Previous reports highlighted an accelerated loss of grey matter associated with cannabis use in schizophrenia [34]. Thus, the contrasting results related to the opposing directions of structural changes in the brainstem and postcentral cortex could be explained as the previous study was conducted in specific participants with adolescent cannabis use onset and schizophrenia. Besides, the increases in postcentral thickness and brainstem volume in our study may possibly demonstrate compensatory hypertrophy or encephaledema after cannabis use.

The fusiform, lateral occipital, postcentral, and brainstem are the brain regions involved in various vital neural activities. Chronic drug abuse can result in toxic organic effects on the brain, which may lead to structural damage [35]. However, the underlying mechanisms of cannabis use on brain structural alterations need to be further investigated. Structural abnormalities in the above regions could serve as early indicators of future functional abnormalities and may contribute to the pathogenesis of neuropsychiatric disorders. To identify the potential patients at an earlier stage, future studies should be conducted to elucidate the association between these structural alternations and neuropsychiatric disorders. Brain MRI, valuable for earlier diagnosis of neuropsychiatric disorders, could also serve as an essential tool for effectively monitoring and implementing preventive strategies for cognitive decline and other neuropsychiatric dysfunctions in cannabis users [36].

It is noteworthy that none of the adjusted P-values reached statistical significance after multiple comparison corrections. While this suggested limited evidence for direct causal relationships, it emphasized the need for cautious interpretation and further investigation using alternative analytical approaches or subgroup analysis to validate these results. Differences in cannabis usage patterns, frequency, and concurrent use of substances like tobacco and alcohol have been reported to exert varying influences on brain structures [37]. As reported in the previous study, cannabis use during late adolescence has been associated with adverse cortical development, particularly in regions abundant in cannabinoid 1 receptors [38]. However, the negative effects of cannabis use during early life may not extend to users of older ages, suggesting differential impacts on brain structures among older users [39]. Besides, current evidence regarding the cognitive effects of long-term cannabis exposure in older adults remains suggestive, with uncertainty about whether cognitive effects revert after cessation [39]. Therefore, the heterogeneity within LCU data used in this study may contribute to inconclusive results, highlighting the need for future research directions focusing on subgroup analyses in these aspects.

Additionally, the choice of atlas was likely to influence the regional findings. Apart from the Desikan-Killiany atlas utilized here, recent efforts have partitioned the cortex into 180 regions using high-resolution multimodal assessments [40]. Other atlases based on functional partitions, particularly for functional MRI data analysis, have also been employed [41]. Furthermore, white matter microstructure, which may involve more pathway-specific genetic influences, may exhibit greater sensitivity to cannabis effects compared to measures of grey matter. Prior research has demonstrated diminished integrity and coherence of white matter in early cannabis users relative to controls [42]. Therefore, investigating genetic influences on the cortex at finer scales, functional levels, and within white matter structures may represent a crucial direction for future research efforts.

In this study, we performed a two-sample MR analysis using high-quality GWAS data with large sample sizes on cannabis use and brain structures. To the best of our knowledge, this is the first study to implement an MR analysis addressing the causal relationship between cannabis use and brain structures. However, this study has several limitations. Firstly, the participants of the GWAS utilized in this study were primarily of European ancestry, which limits the generalizability of our findings to other populations. Secondly, the LCU GWAS relied on self-reported exposure information, which might be affected by recall bias and response bias. Thirdly, specific GWAS data regarding the dose or onset age of cannabis use were not available, making it impossible to develop a degree or age-response relationship. Lastly, to include more instruments in the MR analysis, we relaxed the significance threshold for instrumental variables extraction, which could potentially increase the risk of weak instrument bias and horizontal pleiotropy. However, we conducted a series of sensitivity analyses to mitigate the effects of horizontal pleiotropy.

## Conclusions

In conclusion, while our study did not yield robust and sufficient evidence to support the causal association between cannabis use and brain structures, our findings suggested that four brain regions (fusiform, lateral occipital, postcentral, and brainstem) might exhibit heightened sensitivity in cannabis users. These findings serve as a starting point for further investigation into the connections between cannabis use and other neuroanatomical structures. With the growing popularity of cannabis for both medicinal and recreational purposes globally, further research is essential to enhance our understanding of how cannabis impacts specific brain structures and overall health outcomes.
